# Do Advanced Therapies Have a Future in the Low- and Middle-Income Countries - The Case of Bulgaria, Romania, and Poland

**DOI:** 10.3389/fpubh.2021.729847

**Published:** 2021-08-23

**Authors:** Maria Kamusheva, Adina Turcu-Stiolica, Jakub Gierczyński, Mihaela-Simona Subtirelu, Marcin Czech, Guenka Petrova

**Affiliations:** ^1^Department of Organization and Economics of Pharmacy, Faculty of Pharmacy, Medical University of Sofia, Sofia, Bulgaria; ^2^Pharmacoeconomics Department, Faculty of Pharmacy, University of Medicine and Pharmacy of Craiova, Craiova, Romania; ^3^Researcher Institute of Healthcare Management, Lazarski University, Warsaw, Poland; ^4^Department of Pharmacoeconomics, Institute of Mother and Child, Warsaw, Poland

**Keywords:** advanced therapies, HTA, low- and middle-income countries, recommendations, barriers

## Abstract

**Introduction:** The significant therapeutic potential of the advanced therapies (ATs) has predetermined the increased interests in their development mainly in the context of rare diseases most of which are genetically determined. However, there are still many challenges in front of the health insurance funds related to the cost-effectiveness and budget impact issues of these therapies. Our aim was to review and analyze the potential of low- and middle-income countries for health technology assessment (HTA) of advanced therapies focusing on Bulgaria, Romania and Poland as reference countries. A literature review of the existing good practices related to HTA of advanced therapies across the world and comparison with the national reality were performed. A list of challenges and issues from the point of view of the payer institution of all analyzed countries was performed. Pilot recommendations on how to overcome the barriers were created based on the existing practices and the potential of the national system.

**Discussion:** 15 out of 80 articles identified in PubMed were found as applicable to the study scope as most of them were published in the period 2019–2021. Undoubtedly, the main challenges correspond to the high treatment costs, the uncertainty in clinical effectiveness, and poor HTA methodological approaches applicable for ATs worldwide. The issues identified for low and middle-income countries are similar having as well the lack of enough qualified health economists for the purposes of assessment and appraisal of HTA dossiers of the advanced therapies, lack of adequate existing separate financial programs for those therapies, and not preparedness of the health system and the society as a whole for such therapies.

**Conclusions:** Despite the difficulties and challenges, the advanced therapies can be defined as a futuristic therapy for which great discoveries are yet to come. Therefore, each country should consider the implementation of reliable and nationally oriented programs for HTA and adequate financial coverage of these therapies.

## Introduction

In the recent decades, the expected duration of life has been increased mainly due to the huge number of scientific medical and pharmaceutical advances. The discovery in pathogenesis of many disorders through innovative methods of cell and molecular biology and genetics, new target cell structures and the improvement in the scientific approaches and methods for new drugs allow the development of specific target therapies that give hope for treatment of diseases for which it is not currently available. The significant therapeutic potential of gene therapy, somatic cell therapy and tissue engineering, collectively known as “advanced therapies,” predetermines the growing interest in the development of these products, especially in the field of rare diseases, which in most of the cases are genetically determined. Undoubtedly, the advanced therapy medicinal products (ATMPs) are a fast-growing field with the innovative potential to modify the treatment of a wide range of pathologies not only rare but common oncology, cardiovascular, musculoskeletal and immune diseases etc. for which the conventional approaches are ineffective (1). Despite centralized procedure for marketing authorization of advanced therapies in the European Union (EU), there are still challenges regarding marketing of these products as well as their assessment and appraisal for the purposes of pricing and reimbursement (2). Having the increasing number of approved ATMPs and their high cost, the decision makers need economic evidence with limited level of bias to take the reimbursement decision (3). The challenges related to ATMPs are mainly related with their extremely high prices and the uncertainty in their clinical value in real world settings (2). Their long-term effect is proved but most payer institutions consider only the short-term outcomes and do not pay attention to the significant long-term societal savings (4, 5). Moreover, the ATMPs require specific methods of administration and specialized clinical expert centers for administration and monitoring (5). Many recommendations were given in the scientific literature how to cope with the barriers and challenges such as early dialogue between Health Technology Assessment (HTA) Agencies and other stakeholders, patients' registries in the post-authorization period, outcome-based managed entry agreements, adopt societal perspective in a long-term period etc. (6, 7). The recent proposal for joint HTA increase the authority of European Medicines Agency (EMA) in providing scientific advice in the field and earlier joint creation of evidence that will satisfy the marketing authorization and market access of innovative therapies (8). This will create a challenge in front the middle-income countries due to their limited financial resources in the process of early adoption of ATMPs.

The main objective of this paper is to investigate the potential challenges in front of three middle-income European countries (Bulgaria, Romania and Poland) in health technology assessment (HTA) and decision making for funding the ATMPs.

It is a three steps analytical study. First, we identify articles related to the challenges of ATMPs assessment and employed methodologies for funding, then study all advanced therapies authorized for sale in Europe and at the end analyzed the assessed and funded ATMPs in the countries under consideration. We used Romania, Bulgaria and Poland as reference countries due to their similar level of development and similarities in pricing and reimbursement policies.

A literature review on the practices related to HTA of advanced therapies across the world were performed. A search in the scientific databases such as PubMed and other gray sources (Google Scholar and non-peer review journals) for the last 10 year-period was initiated. The key words for searching process were: gene therapies AND cell therapies AND health technology assessment. In the final analysis HTA agencies reports, scientific papers, recommendations of experts were included and summarized. No restrictions on language and territory of the conducted studies were adopted as eligibility criteria.

All ATMPs authorized in the EU were identified through the publicly available register of the European Medicines Agency (EMA) in the EMA website. The products were systematized according to ATC code, trade name, type of therapy, active substance, indications, date and details of their marketing authorization. Then was performed a search in the Positive drugs lists and official HTA reports issued by the national HTA bodies of the countries under consideration to identify the reimbursed and appraised ATMPs and compared them with the European authorization.

An overview of the current national pricing and reimbursement practices and HTA regulations in Bulgaria, Romania and Poland was conducted and analyzed with focus on the existing requirements for ATMPs assessment.

As a result of the literature review, regulatory analysis and ATMPs autorisation and reimbursement a list of challenges was formulated from the point of view of the payer institution in the observed countries. Recommendations on how to overcome the barriers were created based on the existing practices and the potential of the national systems. Each country was represented by two leading experts with profound experience in reimbursement systems.

## Literature Review

According to Shukla et al. (9) health care systems are not prepared to reimburse treatments with very high prices and related costs for millions of patients. Different views are presented in the literature. According to The National Institute for Health and Care Excellence (NICE) no specific HTA methodology should be adopted for ATMPs (10), while Marsden et al. (5), and Drummond et al. (6) suggested new analytic approaches to estimate value for money. UK and USA differ in opinion whether a new or adopted assessment framework should be implemented for ATMPs. NICE uses their standard HTA, while the Institute for Clinical and Economic Review in the USA published adaptations for the so called “high-impact, single or short-term” therapies. The authors suggest adopting of annuity or amortization of payments over a fixed time period and implementation of innovative outcomes-based performance payments (11–13).

Despite the challenges faced by ATMPs in demonstrating their value the current HTA methodology should be considered having into account also the specific characteristics of ATMPs (14, 15). The standard procedure for pricing and reimbursement is applied in all analyzed countries (Italy, UK, Spain, Frane and Germany) for the ATMPs with some exceptions in Germany. Request for pricing and reimbursement of all authorized in the EU ATMPs has not been submitted in any of the countries analyzed by Ronco et al. (2). Only Chimeric Antigen Receptor-T cells (CAR-Ts) are reimbursed in all analyzed Western countries. Outcome-based Managed Entry Agreements, accreditation process of hospitals for ATMPs management, extra-funds for hospitals, higher incremental cost-effectiveness ratio (ICER) thresholds and additional fund per episode are some of the specific methods used in some of the countries (2).

Uncertainty in the value of gene therapies and in their long-term benefits, limited clinical results, high costs, challenges in conduction of conventional randomized clinical trials (RCTs), limited number of included in the RCTs patients, heterogeneity of the patient population, lack of adequate comparators put a lot of questions in front the decision makers. Marsden et al. (5) gives a number of challenges and related recommendations to manufacturers and payer institutions how to cope with them. The paucity of long-term clinical trial data is one of the main issues in the process of performing cost-effectiveness (CEA) and cost-utility analysis (CUA) (16). Therefore, modification of the economic evaluation approach is required, for ex. two perspectives to be considered in cost-effectiveness analysis—the perspective of the society because of the expected long-term benefits from gene therapies and the perspective of the health insurance fund. Considering specific characteristics of ATMPs, Angelis et al. (16) developed a set of methods for adaptation and improvement of the traditional economic evaluations highlighting the importance of long-term evidence-based results. Application of CUA and QALY methodology to ATMPs is commented with some concerns. So, SAVE (saved young life equivalents) is suggested as a preferred method to value lifetime health profiles of curative treatments such as gene therapies. Cost-benefit analysis could be also used as beneficial alternative for assessment of ATMPs (17). Type of health system should be also considered—whether it is a private or public, multi-, or single payer. Drummond et al. (6) highlighted those innovations in payment systems that are required to correspond to any developments in the HTA methods for gene therapies and recommends a separate checklist for economic evaluations of gene therapies: the severity of the disease, unmet medical needs, type of conducted clinical trials, overall value of the therapy to the caregivers, to the healthcare system and patients. Moreover, involvement of patients is a key element in the whole process of HTA for ATMPs (18). Jorgensen et al. emphasized the need of generation real-world evidence for reduction of the uncertainty as well as implementation of outcomes managed entry agreements combined with annuity-based payments to reduce the burden of ATMPs on the budget (19, 20). Driscoll et al. concluded that collecting of real-world evidence and implementation of managed entry agreements based on the outcomes could enhance the possibility for market access of ATMPs.

In the EU, despite adoption of centralized marketing authorization procedure according to the Regulation 1394/2007 as an obligatory one for ATMPs, the real market access differs significantly across different EU countries and sometimes across regions in the same country (21, 22). Therefore, as Driscoll et al. recommends that a common unified approach based on early dialogue between the EU countries Germany, UK, France, Spain, and Italy could be a solution. Similar approach could be developed among Central and Eastern European Countries (CEEC) with identical financial and economic issues (12). More challenges and barriers exist for lower income countries to apply HTA for ATMPs and so called transformative or curative innovations due to lack of enough experience and still existing issues in the assessment of “standard” therapies (23). Lloyd-Williams and Hughes ssummarized the available economic evaluations of ATMPs and performed a critical appraisal of these evaluations highlighting the challenges facing by pharmacoeconomists when analyzing ATMPs (24) (Table 1).

**Table 1:** Recommendations for HTA of advanced therapies—a literature review results.

**References**	**Region/country**	**Challenges**	**Methodology for HTA**
Ronco et al. (2)	UK Italy Spain France Germany	Uncertainty on the value; High average cost per patient; One-shot nature.	•Application of traditional methodology in Italy, Spain, UK, France; •In the UK a Single Technology Appraisal or the Highly Specialized Technologies Programme is applied (for ultra-rare diseases, higher ICER threshold); •In France patient access to a new product is possible before marketing authorization with the Authorization for Temporary Use (ATU); •In Italy—dedicated funds and access to drug lists in case of innovativeness; •Outcome-based managed entry agreements; •Separate Drug Fund (such as Cancer Drug Fund in England); •Extra-cost coverage systems to support the management of ATMPs in hospitals; •Patients access scheme to ATMPs* through the accreditation criteria for center selection.
Drummond et al. (6)	–	Uncertainties about long-term benefits; Limit capability of the economic model to deal with uncertainty.	•Performance-based risk-sharing arrangement; •To perform CEA from 2 perspectives: the societal and the payer's; •Incorporation of broad spectrum of criteria: severity of disease, substantial improvement in life expectancy, lack of other options; •Higher ICER threshold (in England £100 000–£300 000/QALY depending on the QALYs gained); •Longer time horizon in economic evaluation considering more costs and benefits; •Using different discount rates for costs and benefits (the benefits are experienced immediately not in a future period).
Bignami et al. (18)	–	–	•Patients to have a role in health technology assessment.
Driscoll et al. (21)	–	High costs; Restricted budgets; Uncertainty in clincial effectiveness; Absence of supporting long-term data.	•Performance based managed entry agreements; •Post-launch evidence generation; •Additional factors: size of the target population, disease burden and level of unmet need; •Shaping European Early Dialogues for health technologies program—early dialogue between HTA agencies of the EU countries.
Champion et al. (11)	–	Lack of long-term outcome data; Not suitable surrogates for outcomes; High price.	•Higher threshold; •Separate fund; •Change in discounting levels.
Faulkner et al. (23)		Uncertainty regarding efficacy, safety and long-term effect; Uncertain population or subpopulation data, study quality, and uncertainty around cost-effectiveness estimates.	•New evolution and payment models; •Flexible and iterative models; •Novel stakeholder partnerships; •Risk-sharing agreements, convergence of registries, increased emphasis on value-based care.
Jørgensen et al. (19)	UK Italy Spain France Germany	Uncertainty in the results from clinical trials; High costs; Related high costs for administration and additional services.	•Specific funding mechanisms for new and more expensive therapies; •Outcomes modeling and risk-sharing agreements; •To target small population; •Real-world evidence generation.
Lloyd-Williams and Hughes (24)	–	Significant uncertainty and high likelihood of bias; Largely unknown long-term outcomes; A paucity of evidence on health state utilities and extensive modeling assumptions; Value attributes may not be captured adequately in the quality-adjusted life year (QALY); Lack of evidence on utility values.	•Make assumption in models and economic evaluations; •Apply higher discount rate for costs; •Outcomes-based payment; •Critically measurement of therapeutic success in real-world settings.
Jönsson et al. (14)	–	Increased uncertainty in evidences; Levels of discount rates.	•Collection of follow-on real-world evidence via registries; •Developing new methods; •Use of value of information analysis; •Outcomes-based contracts; •Criteria for identifying an innovative product; •Consideration of other aspects of value; •Collecting more evidence to assess whether additional evidence would reduce the uncertainty; •Establishment of an international, multi-disciplinary forum to consider the economic, social and ethical implications of the choice of differential or joint discount rates for costs and benefits; •Broadening the definition of “value.”
Coyle et al. (15)	-	Limitations in the evidence generation; Budget constraints.	•NO FULL TEXT
Shukla et al. (9)	–	High prices; Limited evidence about the safety and effectiveness.	No recommendations are given.
Angelis et al. (16)		Uncertain clinical benefits; Long-term uncertainty.	•To recalibrate the current HTA methods; •Implementation of a set of technical adaptations and methodological refinements; •Alternative financing mechanisms.
Aballéa et al. (17)	-	Limited clinical data; Lack of experience; Methodological concerns for HTA.	•Incorporation of broader elements of value; •Assessment of clinical effectiveness; •Selection of appropriate discount rates; •Prospective registries; •The importance of expert opinion; •Higher cost-effectiveness threshold; •The use of cost-benefit analysis and saved young life equivalents (SAVE) were proposed asalternatives to QALYs; •Multiple Criteria Decision Analysis.
Jørgensen et al. (20)	France, Germany, Italy, Spain, and the UK	Uncertainty around the real-world value of the therapies (potentially lifelong health benefit supported by shorter-term data at launch); Highprices; Affordability challenges.	•Reimbursement on the condition of collecting additional data and subject to future reassessments; •Rebates or staged payments linked to individual patient outcomes (outcomes-based staged payments); •Real-world evidence (RWE) has become an increasingly powerful lever for demonstrating the value of health benefits in the clinical setting.
Marsden et al. (5)	–	Difficulties to generate robust clinical evidence needed by decision-makers; Uncertainty regarding clinical outcomes; Budget constraints; Extremely high up-front prices.	•Collaboration on further policy development to create a stable pricing, financing, andhealth insurance structure; •Negotiating lower prices throughdiscounts or rebates; •Restricting eligible populations; •Requiring patients to try other treatments first before receiving coverage for the preferred option; •Outcomes-based agreements; •Dialogue with payers and regulators; •Post-approval studies to reduce residual uncertainty about the Safety and effectiveness of new therapies; •Criteria for designation of potential centers of excellence for the delivery ofthe therapy.

*ATMPs, advanced therapy medicinal products.

## National Pricing, Reimbursement, and HTA Practice in Romania, Bulgaria and Poland

**Figure 1:**
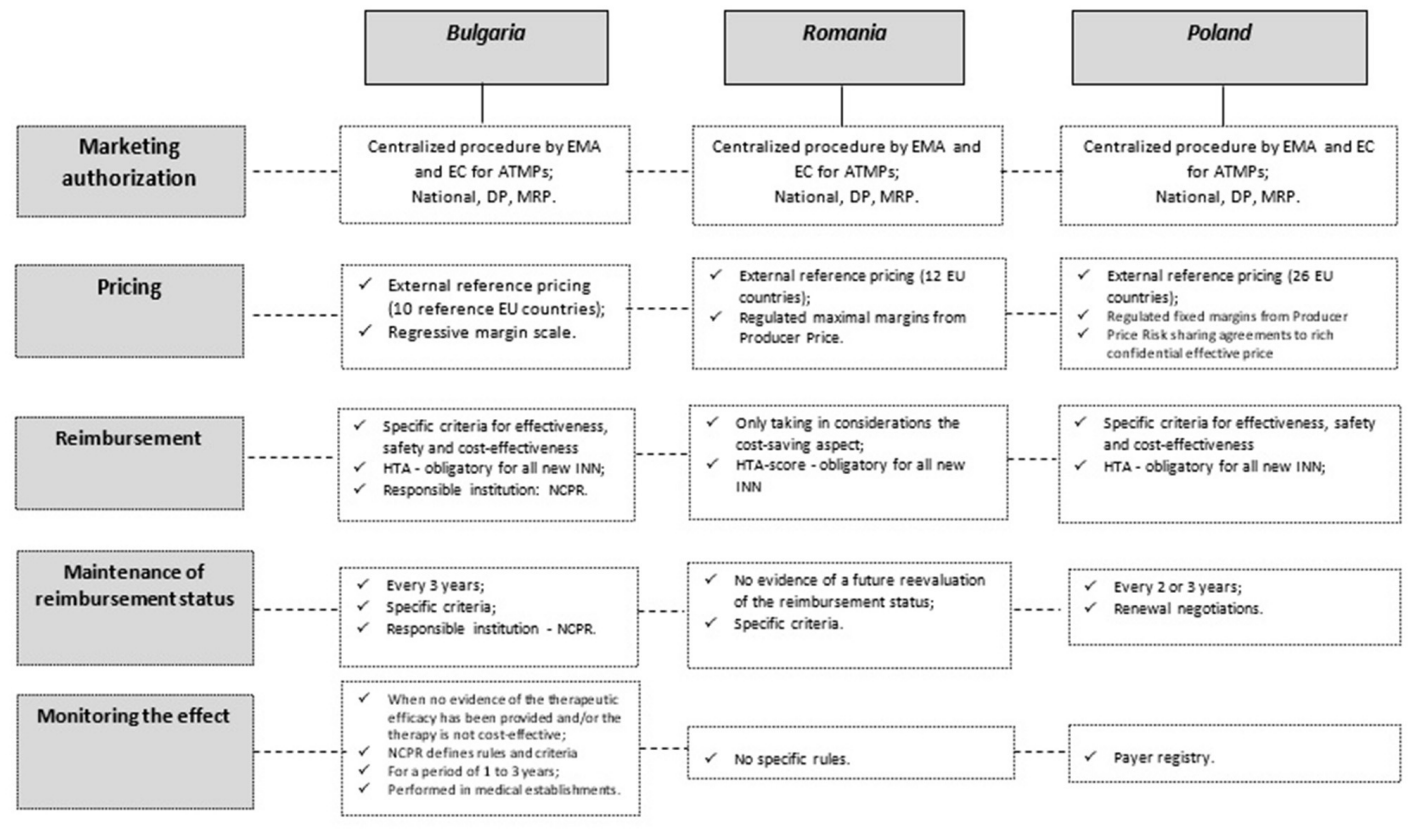
Schematic view of the medicines access to the market in Bulgaria, Romania and Poland. ATMPs, Advanced therapy medicinal products; DP, Decentralized procedure; EC, European Commission; EMA, European Medicines Agency; EU, European Union; HTA, Health Technology Assessment; INN, International non-proprietary name; MRP, Mutual recognition procedure; NCPR, National Council on Prices and Reimbursement of Medicinal Products.

The National Council on Pricing and Reimbursement (NCPR) in Bulgaria was created in 2012 as an independent regulatory body under the governance of the Ministry Council responsible for regulation of prices, and inclusion/exclusion of medicines in the Positive Drug List (PDL). Pharmacoeconomic analysis is required for the purposes of reimbursement. It was performed based on specific methodological approach developed by Petrova and Getov (25). Currently, the HTA legislation in Bulgaria is defined in the Regulation on prices of medicinal products (MPs). The Health Technology Assessment is a part of the procedure for inclusion of MPs in the PDL, consisting of a clinical (efficacy and safety), pharmacoeconomic evaluation and ethical considerations for unmet health care needs. It includes analysis of the health problem, comparative analysis of the therapeutic efficacy, effectiveness and safety, analysis of pharmaco-economic indicators, analysis of the impact on the budget. HTA dossier is prepared for every MPs belonging to a new INN as well as for those already in the PDL but for which marketing authorization holder (MAH) wants to receive extension of the therapeutic indications. At least one positive decision of the HT issued by HTA body in UK, France, Germany and Sweden is required. Ministry of Health and National Health Insurance Fund (NHIF) could also initiate an evaluation of the health technologies for medicinal products already included in the PDL (Figure 1) (26, 27). There is no specific cost-effectiveness threshold stated in the regulation although some recommendation for incremental cost-effectiveness ratio below 3 times GDP is suggested in the corresponding guideline. In addition, there is no specific procedure for ATMPs assessment although the NCPR might request a monitoring of the therapeutic effect in real life practice to ensure the generation of additional product value evidence. There has been a significant development in Bulgarian pharmaceutical legislation regarding the implementation of pharmacoeconomic assessment for the purposes of the inclusion of medicinal products in the national insurance list for the last 20 years but still lot needs to be done in order to ensure to the Bulgarian patients access to innovative and advanced therapies.

In Romania, public health care is provided by the state and is financed from the state budget, local budgets and the National Health Insurance Fund; however, joint payment from patients may also be encountered in some cases. Romania had a pricing model for the first time in 2009, during the financial crisis, when several measures have been taken to keep the expenditure of medicines under strict control. Prices have been published in the public catalog of medicines called Canamed and are considered the maximum prices allowed in Romania for medicinal products (28).

The implementation of the HTA in Romania began in 2012, and an important thing that accelerated the implementation process was the need to adopt the EU Directive 2011/24/ (29) on the application of patients' rights in health legislation in Romanian legislation. The HTA structure has become a department within the National Agency for Medicines and Medical Devices (NAMMD) in 2014. After that, several evaluation criteria were introduced (30). The HTA scoring system in Romania is active and the prescription of medicines is on INN's (only biological medicines must be prescribed under the brand name) (31). The government has implemented only the cost of volume and the cost of volume—results from contracts in the risk-sharing mechanism. The inclusion of a new INN on the reimbursement list is subject to prior assessment by the NMMDA during the health technology assessment procedure. Such an evaluation procedure must be based on the following criteria:

- health technology assessment reports from authorized agencies in France, Germany and the United Kingdom;
- the necessary data required to calculate the costs of therapy;
- the summary characteristics of the products,
- compensation status in the EU;
- the price approved by the MoH;
- a document stating the intention of the relevant marketing authorization holder to engage in a cost-volume or cost-volume-result mechanism where the individually calculated score corresponds to conditional inclusion on the reimbursement list.

In Poland reimbursement decisions are made by the Ministry of Health on the provision of Reimbursement Act from 2012 (32). The Ministry of Health is responsible for deciding which medicines will be reimbursed and how they will be priced. The Ministry of Health is advised by the Drug Policy and Pharmacy Department and Economic Committee, which process reimbursement applications and negotiate prices. Medicines are chosen to be reimbursed based on the product's efficacy in treating disease and their cost. Health Technology Assessment Agency evaluates all new molecules. Cost-effectiveness threshold is now on the level of 155 thousand PLN (35 thousand EUR) per QALY. There is no exception from the use of cost/QALY for rare and oncological diseases. In general, medicines with excessive prices or limited efficacy are not reimbursed. Prices are compared to 26 European Union countries. Most expensive medicines are reimbursed on the basis of drug programs, which are designed for defined group of patients, with precisely defined inclusion/exclusion criteria and careful monitoring. The Medical Fund Act which came into power in 2020 is a new presidential initiative with an objective to improve care in oncology and rare diseases (33). Within Medical Fund oncology and orphan medicines registered from 1.01.2017 till 26.11.2020 can be reimbursed, for which producers did not submit applications for public reimbursement to the Minister of Health. The first list of highly innovative medicines registered in European Union from 1.01.2020 till 26.11.2020 was publish by HTA Agency on 26.02.2021 (34). The list contained 11 oncology and orphan drugs—including advanced therapy representative—Zolgensma®. The Minister of Health selected 5 drugs from this list (including Zolgensma) with an objective of to price negotiations with manufacturers and eventually to issue reimbursement decisions. Second list of drugs with high clinical value is planned to be published on 26.08.2021.

All currently authorized in the EU ATMPs are presented in Table 2. Most of them are gene therapies or cell-based gene therapies such as Luxturna (virus that contains normal copies of the RPE65 gene), Strimvelis (gene for ADA is inserted into the CD34+ cells using retrovirus), Zynteglo (stem cells modified by a virus that carries copies of the beta-globin gene), Kymriah, Yescarta **(**T cells that have been modified genetically), Zolgensma (contains a functional copy of SMN1 gene), Imlygic. The other three therapies are cell therapies: Alofisel (mesenchymal stem cells from the fat tissue of a donor), Holoclar (limbal cells, which include cells from the surface of the cornea and limbal stem cells grown in a laboratory) and Spherox (contains spheroids). Zalmoxis has been withdrawn at the request of the marketing authorization holder in 2019 (37).

**Table 2:** Advanced therapies authorized in the European Union (35, 36).

**ATC code**	**Trade name**	**Type of therapy**	**Active substance**	**INN**	**Date of marketing authorization**	**Indications**	**Marketing authorization details**
M09AX09	Zolgensma	Gene therapy	Onasemnogene abeparvovec		18/05/2020	Muscular Atrophy, Spinal	Under additional monitoring; Conditional marketing authorization; Designated an orphan medicine.
B06A	Zynteglo	A cell-based gene therapy	Autologous CD34+ cell enriched population that contains hematopoietic stem cells transduced with lentiglobin BB305 lentiviral vector encoding the beta-A-T87Q-globin gene	Betibeglogene autotemcel	29/05/2019	Beta-Thalassemia	An accelerated assessment; Under additional monitoring; Received a conditional marketing authorization; Designated an orphan medicine.
L01XX71	Kymriah	A cell-based gene therapy	Tisagenlecleucel		22/08/2018	Precursor B-Cell Lymphoblastic Leukemia-Lymphoma; Lymphoma, Large B-Cell, Diffuse	Under additional monitoring; Designated an orphan medicine.
Not yet assigned	Luxturna	Gene therapy	Voretigene neparvovec		22/11/2018	Leber congenital amaurosis Retinitis pigmentosa	Under additional monitoring; Designated an orphan medicine.
L04	Alofisel	Cell therapy	Darvadstrocel		23/03/2018	Rectal Fistula	Under additional monitoring; Designated an orphan medicine.
L01X	Yescarta	A cell-based gene therapy	Axicabtagene ciloleucel		23/08/2018	Lymphoma, follicular Lymphoma, Large B-cell, Diffuse	Under additional monitoring; Designated an orphan medicine.
M09AX02	Spherox	Cell therapy	Spheroids of human autologous matrix-associated chondrocytes		10/07/2017	Cartilage diseases	Under additional monitoring.
L03	Strimvelis	A cell-based gene therapy	Autologous CD34+ enriched cell fraction that contains CD34+ cells transduced with retroviral vector that encodes for the human adenosine deaminase (ADA) cDNA sequence from human haematopoietic stem/progenitor (CD34+) cells	Autologous CD34+ enriched cell fraction that contains CD34+ cells transduced with retroviral vector that encodes for the human ADA cDNA sequence	26/05/2016	Severe combined immunodeficiency	Under additional monitoring; Designated an orphan medicine.
L01	Zalmoxis	Cell therapy	Allogeneic T cells genetically modified with a retroviral vector encoding for a truncated form of the human low affinity nerve growth factor receptor (ΔLNGFR) and the herpes simplex I virus thymidine kinase (HSV-TK Mut2)		18/08/2016	Hematopoietic stem cell transplantation Graft vs. host disease	The marketing authorization for Zalmoxis has been withdrawn at the request of the marketing authorization holder in 2019.
L01XX51	Imlygic	Gene therapy	Talimogene laherparepvec		16/12/2015	Melanoma	
S01XA19	Holoclar	Cell therapy	*Ex Vivo* expanded autologous human corneal epithelial cells containing stem cells		17/02/2015	Stem cell transplantation Corneal diseases	Under additional monitoring; Conditional marketing authorization; Designated an orphan medicine.

In Bulgaria, there is only one prepared and published HTA report for advanced therapy (Alofisel®). The decision issued by the NCPR to include Alofisel in the reimbursement list was negative. Cost-benefit analysis for the population of patients with fistulizing Crohn disease showed that the costs for administration of darvadstrocel outweigh the benefits, reported as avoided costs. The results of the cost-benefit analysis show that its application leads to quality adjusted life-years gained and to increased costs for the healthcare system (38). Nusinersen for spinal puscular atrophy (ATC code M09AX07), which is a type of gene therapy (survival motor neuron (SMN)-inducing therapy or anti-sense oligonucleotide medicine), but not classified as an advanced therapy, was included in the Positive Drug List in Bulgaria in 2019. More than 7 million euros was paid for Nusinersen treatment by the NHIF in 2020 (39).

Only two ATMPs were evaluated by HTA body in Romania: Kymriah and Alofisel to the current date. Kymriah has a positive HTA report in Romania issued in 02.04.2020. The final decision of the HTA Department was to include the drug with DCI Tisagenlecleucel for the following indication: “treatment of adult patients with diffuse, large B-cell lymphoma, recurrent or refractory, after two or more lines of systemic therapy” in the list, containing the common international names corresponding to medicine to which they benefit insured patients, on the basis of a medical prescription, in the social insurance system of health, sublist C-section C2- P3 “National oncology program” (40).

In Poland three ATMPs were evaluated by HTA Agency: Kymriah®, Yescarta® and Zolgensma®. Kymriah received HTA Agency negative recommendation on 30.01.2021 (41) and Yescarta received negative recommendation on 12.03.2021 (42). The President of the HTA Agency, taking into account the criteria in line with the assessment of medical technologies, i.e., the size of the health effect obtained, cost effectiveness and the projected impact on the payer's budget, as well as the importance of the health problem and the uncertainty of estimates, considered it unjustified to finance from the public funds based on Kymriah and Yescarta so far proposed conditions. Zolgensma was assessed positively by HTA Agency on 26.02.2021 as an technology with high degree of innovation within the Medical Fund and directed to further price negotiations on the Ministry of Health level (43).

Kymriah is reimbursed in 14 other EU states including Austria, Belgium, Czech Republic, Croatia, Finland, France, Germany, Greece, Italy, Luxembourg, Portugal, United Kingdom, Slovenia, Spain. Alofisel dossier has been submitted for evaluation in HTA department on 02.05.2019 and the drug with the DCI Darvadstrocel is reimbursed in 3 member states of the European Union: Austria, Finland, the Netherlands. The recommendation of the Department of HTA in Romania for Alofisel is the development of the therapeutic protocol for the drug having the indication “treatment of complex perianal fistulas in adult patients with non-active/slightly active luminal Crohn's disease, then when the fistulas show an inadequate response to at least one conventional or biological treatment” (44). Both decisions had a Budget impact analysis (BIA) focusing on the direct costs, the HTA decisions from France, Germany and UK and the number of EU countries (27) in which the drug is reimbursed (45).

## Challenges and Recommendations for HTA of ATMPS in Bulgaria, Romania and Poland

Considering the specific procedures for Health technology assessment in the analyzed countries and the approved and implemented approaches for appraisal of new medicines, we identified the possible challenges (Table 3). They are separated in 3 main groups: economics/pharmacoeconomics issues, clinical, and technical/administrative barriers. Undoubtedly, the most serious barrier is related to the extremely high prices of advanced therapies and budget constraints. Uncertainty in the clinical efficacy due to specifics of ATMPs are consequences mainly of the clinical trials design and limited number of included patients.

**Table 3:** Main challenges for reimbursement of ATMPs in Bulgaria, Romania, and Poland.

	**Pharmacoeconomics and economics issues**			**Clinical issues**	**Technical/administrative issues**				
	**High prices**	**High cost-effectiveness ratio**	**Restricted budgets**	**Low level of evidences/uncertainty in the available evidences**	**Lack of/or not enough centers for excellence and experts**	**Lack of specific methodological criteria for HTA of ATMPs**	**Limited possibilities for follow-up through prospective electronic registries**	**Requirements for reimbursement of the health technology-candidate by other health insurance funds**	**Requirements for at least 1 positive opinion for reimbursement by HTA agency of other countries**
Bulgaria	+	+	+	+	+	+^*^	+	+	+
Romania	+	+	+	+	+	+^*^	+	+	+
Poland	+	+	+	+	+	+	+	–	–

* *Specific requirements for orphan medicines exist in the methodology for HTA*.

Framing the recommendations is based not on the results of special designed survey among key opinion leaders but on the critical analysis of the performed literature review, national legislative documents and the HTA experience of the authors. The national systems posses already an experience with discounts, rebates and application of manage entry agreements for traditional medicines. Adapting of specific texts in the legislative documents for implementation of outcome-based management entry agreements, development of effective e-health registries for follow-up the patients on treatment with ATMPs, determining a higher threshold etc. could be considered (Table 4).

**Table 4:** Recommendations for overcoming the barriers.

	**Pharmacoeconomics and economics issues**			**Clinical issues**	**Technical/administrative issues**				
	**High prices**	**High cost-effectiveness ratio**	**Restricted budgets**	**Low level of evidences**	**Lack of/or not enough centers for excellence and experts**	**Lack of specific methodological criteria for HTA of ATMPs**	**Limited possibilities for follow-up through prospective electronic registries**	**Requirements for reimbursement of the health technology-candidate by other health insurance funds**	**Requirements for at least 1 positive opinion for reimbursement by HTA agency of other countries**
Bulgaria	Rebates, outcomes based MEAs	Determing of higher threshold for ATMPs	Separate fund covered by the governement; Eligibility criteria for treatment	Collection of additional evidences on the basis of follow-up data	Financial and administrative	Adapting and improving of the current methodology	Extensive development of e-health services at all levels	Adopting of the legislative requirements	Adopting of the legislative requirements
Romania	Rebates	Determing of higher threshold for ATMPs	Eligibility criteria for treatment	Collection of additional evidence	Creating special academic institutions	Improving the current methodology	Development of e-health services	Modification of the current legislation	Modification of the current legislation
Poland	Rebates, outcomes based MEAs	Determing of higher threshold for ATMPs	Restricted populations and budget availability from potential savings	Collection of additional evidences on the basis of follow-up data	Certification of centers of excellence	Adapting and improving of the current methodology	Development of e-health services—specially payer registries	Adopting of the legislative requirements	Positive opinion of HTA Agency in Poland

## Discussion

Many studies about advanced therapies have been published in the recent years. The barriers for health technology assessment of ATMPs and a number of measures how to overcome them are widely discussed especially for Western European countries and USA (2, 5–24). All of them identified as main and significant obstacles the extremely high prices of the advanced therapies and the uncertainty about their clinical effect in a long-term period. Additional barriers highlighted by the authors are related to inadequate existing methodology for economic and pharmacoeconomic evaluations—QALY concept, extrapolation of the benefits, application of biased models based on too many assumptions with limit capability to deal with uncertainty and comparison with the conventional threshold are pointed out as not suitable ways for assessment of the new advanced therapies (6, 16, 17).

The authors proposed various, in most articles overlapping suggestions how to deal with the obstacles: adopting of new methodology for HTA of ATMPs, implementation of innovative outcomes-based performance payments, change in the discounting rates, collecting of real-world evidence and not applying cost-utility but cost-benefit analysis from the broader societal perspective due to the expected long-term benefits of the advanced curative therapies (17, 21, 22).

Each country should specify its own methodology on the basis of its health system financing, current practice and available best practices worldwide. While some Western European countries apply the traditional HTA methodology, others have implemented specific appraisal or highly specialized programs for ultra-rare diseases and higher ICER threshold. Outcome-based managed entry agreements are preferable options as well as creation of separate drug fund for overly expensive medicines (2). The Eastern European Countries as Bulgaria, Romania, and Poland face not only financial but also organizational and technical issues related to establishment of adequate centers for excellence responsible for storing and application of these specific therapies; as well as lack of systematized and working e-health registers for follow-up and collection of long-term results for safety and efficacy of the advanced therapies. With the aim to protect the public funds are endorsed some administrative barriers like the positive HTA assessment in at least one country or reimbursement of the product in five other countries as is the case of Bulgaria. This creates a barrier in front the market entrance of ATMPs on the national market. In Poland price comparison of a particular medical technology between EU countries is crucial—with focus on the lowest price.

From all ATMPs authorized by the European Commission based on the scientific assessment of the European Medicines Agency, only three were assessed in Poland (Kymriah®, Yescarta®, and Zolgensma®), two of them have been assessed in Romania (Kymriah®, Alofisel®) and one in Bulgaria (Alofisel®). So, there is still limited or lack of experience in all three countries in the field of HTA of ATMPs which probably gives one step ahead for furthermore extensive attempts to develop and implement working mechanisms based on the best practices and local specificities.

The barriers for HTA of ATMPs and recommendations for overcoming the barriers are formulated only on the basis of the key opinion leaders opinion which could be defined as a strong limitation of the current study. Further analytical and comparative studies among countries focused on the HTA concerns of ATMPs should be performed and compared with the current developed lists with issues and suggestions.

## Conclusions

Our situation analysis shows that the selected countries from Central and Eastern Europe have not developed adequate and specific methods for assessment and appraisal of advanced therapies. Their capacity is limited and has appraised few of these therapies in the recent years. Despite the difficulties and challenges, the advanced therapies can be defined as a futuristic therapy for which great discoveries are yet to come. More and more ATMPs are expected to be developed and to be potential candidates for reimbursement. Therefore, each country especially low- and middle-income countries should consider the implementation of reliable and nationally oriented programs for HTA and adequate financial coverage of these therapies. Many challenges exist which requires deep and detailed discussions and analyses of the current situation so as the most suitable solutions to be found and implemented.

## Author Contributions

MK, AT-S, and MC carried out the research and drafted the manuscript. MK and GP performed the literature review. MK, GP, AT-S, M-SS, MC, and JG participated in the study design and reviewed the paper. All the authors have provided valuable contributions to the manuscript, read, and approved the final manuscript.

## Conflict of Interest

The authors declare that the research was conducted in the absence of any commercial or financial relationships that could be construed as a potential conflict of interest.

## Publisher's Note

All claims expressed in this article are solely those of the authors and do not necessarily represent those of their affiliated organizations, or those of the publisher, the editors and the reviewers. Any product that may be evaluated in this article, or claim that may be made by its manufacturer, is not guaranteed or endorsed by the publisher.
